# *In Vitro* Wound Healing Activities of Three Most Commonly Used Thai Medicinal Plants and Their Three Markers

**DOI:** 10.1155/2020/6795383

**Published:** 2020-06-29

**Authors:** Metar Siriwattanasatorn, Arunporn Itharat, Pakakrong Thongdeeying, Buncha Ooraikul

**Affiliations:** ^1^Applied Thai Traditional Medicine Faculty of Medicine, Thammasat University, Khlong Luang, Pathum Thani 12120, Thailand; ^2^Department of Applied Thai Traditional Medicine and Centre of Excellence on Applied Thai Traditional Medicine Research (CEATMR), Faculty of Medicine, Thammasat University, Khlong Luang, Pathum Thani 12120, Thailand; ^3^Department of Agricultural Food and Nutritional Science, Faculty of Agricultural Life and Environmental Sciences, University of Alberta, Edmonton, Alberta T6G 2P5, Canada

## Abstract

Skin ensures that a constant internal environment can be maintained in an ever-changing external environment. When a wound occurs on the skin, the inflammatory and proliferative phases are initiated in response to injury. Thai traditional medicine (TTM), using medicinal plants and ancient knowledge, has been used to treat wounds. Eight Thai medicinal plants, most commonly used to treat wounds, were evaluated for their *in vitro* biological activities such as antioxidation by NBT assay, anti-inflammation by production inhibition of NO, promoting fibroblast cell proliferation, and wound closure activities. Plant materials were extracted with 95% ethanol or distilled water and then concentrated and dried. Statistical analysis of data was done using one-way ANOVA at *p* value of 0.05. The ethanolic extracts of *Garcinia mangostana* L., *Glycyrrhiza glabra* L., and *Nigella sativa* L. could inhibit the production of superoxide anion with the IC_50_ values of 13.97 ± 0.38, 28.62 ± 1.91, and 71.54 ± 3.22 *μ*g/ml and nitric oxide with the IC_50_ values of 23.97 ± 0.91, 46.35 ± 0.43, and 78.48 ± 4.46 *μ*g/ml, respectively. These extracts could promote cell proliferation and accelerate wound recovery at the rate of 2.02 ± 0.03, 2.12 ± 0.03, and 2.65 ± 0.05% per hour, respectively. Three established markers from these three plants were selected according to the selection criteria. Alpha-mangostin, glycyrrhizin, and thymoquinone were found to be the active markers for wound closure activities. The ethanolic extracts of *G*. *mangostana*, *G*. *glabra*, and *N*. *sativa* could scavenge superoxide anion and inhibit the production of nitric oxide; therefore these extracts could assist in surpassing the inflammatory phase and protected the cells surrounding the wound area. Most importantly, these extracts also increased the proliferation and accelerated wound closure, indicating that these plant extracts could be promoting wound healing processes and support the use of TTM.

## 1. Introduction

The skin forms a barrier that protects the body from pathogens, maintains moisture, and preserves the temperature of the body. A wound is a type of injury on the skin which causes physical, chemical, electrical, or thermal damage. When a wound occurs, the healing process is immediately initiated in response to the injury to regenerate the tissue. Wound healing consists of 3 phases, that is, inflammation, proliferation, and remodelling, a complex process associated with multiple cells, and molecules [1]. When a wound occurs on the skin, within 10–30 minutes the inflammatory phase is activated which induces vasodilation and increases capillary permeability that causes pain, swelling, and redness at the wound area. When the immune cells such as neutrophils and macrophages reach the wound, nitric oxide (NO) as the primary reactive nitrogen species (RNS) was produced to destroy the cell debris and bacteria. Nitric oxide is formed from L-arginine and molecular oxygen (O_2_) by the enzyme nitric oxide synthase (NOS). Additionally, the primary reactive oxygen species (ROS) or the superoxide anion radical (O_2_^−^) are formed from molecular oxygen (O_2_) during cellular metabolism in the mitochondrial electron transport chain (METC) and others [2]. Nevertheless, increments of ROS and RNS cause damage to nucleic acids, proteins, and lipids of cells surrounding the wound leading to loss of functions and cell death [3]. When the inflammation phase is passed and all the potentially dangerous factors are removed, the proliferation phase commences. Fibroblasts play a major role in this process. Fibroblasts, differentiated into myofibroblasts, mainly via the activation of mediators, migrate to the wound area and produce abundant extracellular matrix [4]. Myofibroblasts also express smooth muscle actin filaments in order to facilitate wound contraction and achieve skin restoration while epithelial cells migrated to cover tissue from the edge of the wound. The remodelling phase is the final phase of the wound healing process, which begins about 20 days after the wound occurs on the skin. From months to years, this period consists of the collagen cross-link and different cells of the wound; then the creation of collagen decreases until the balance is achieved.

Thai traditional medicine (TTM) is based on Thai ancient knowledge that has been compiled into various scriptures through experiences and observations by traditional doctors and passed onto the next generations. The list of medicinal plants most commonly used to treat wounds was selected from the Mukkharoka scripture, the National List of Essential Medicines of Thailand, and traditional usages. From the above criteria, the seed of black cumin (*Nigella sativa* L.) appeared in most of the recipes in Mukkharoka scripture for the treatment of wound and inflammation in oral cavity. Previous studies found that *N*. *sativa* and its major compound thymoquinone have been shown for their therapeutic benefits such as antibacterial, antioxidant, and anti-inflammatory activities [5–7]. The root of licorice (*Glycyrrhiza glabra* L.) was found as the main ingredients of Am-ma-ruek-ka-va-tee recipe while dry fruits of Indian nightshade (*Solanum indicum* L.) and dry fruits of Thai nightshade (*Solanum trilobatum* L.) were found as the main ingredients of Phra-sa-ma-vaeng recipe in the National List of Essential Medicines of Thailand. These medicines were used to treat sore throat, cough with sputum, and ailments in the oral cavity. The major compounds of these extracts had been proved to be glycyrrhizin (*Glycyrrhiza glabra*) and steroidal glycoside (solanidine and solasodine in *Solanum* spp.) respectively, with reported activities such as antibacterial, anti-inflammatory, antioxidant, and anticancer activities [8–10]. Indian gooseberry (*Phyllanthus emblica* L.) fruits were found as the main ingredient in Tri-pha-la recipe; TTM used this recipe for symptoms in the upper respiratory tract and to balance the internal body. Gallic acid, protocatechuic acid, and tannins were found in its extract with bioactivities such as anti-inflammatory, antioxidant, and analgesic activities [11, 12]. The pericarp of mangosteen (*Garcinia mangostana* L.) had traditional use such as wound cleansing liquid on the skin and feet. The major compound of *G*. *mangostana*, *α*-mangostin, had various proven bioactivities such as antioxidant, antibacterial, antifungal, anticancer, and anti-inflammatory activities [13]. The bark of *Mimusops elengi* L. was used for toothache; it consisted of many compounds such as steroids, flavonoid, terpenoids, glycoside, saponin, and tannin with bioactivities such as antioxidant and anti-inflammatory activities [14, 15]. Clove bud (*Syzygium aromaticum* L.) has been used in throat lozenge for pain relief and its major active compound is eugenol; therefore *S*. *aromaticum* extract many bioactivities such as antioxidant and antihyperglycemic activities [16–18].

Though TTM has long been used to cure various diseases, it lacks scientific descriptions and evidence common to modern medicine. Therefore, to make it more relevant and acceptable to general healthcare practitioners and patients, it is necessary to find out scientific evidence that would help describe its activities and demonstrate its efficacy. This study focused on eight Thai medicinal plants for their bioactivities associated with anti-inflammatory and antioxidant activities and the acceleration of wound healing as well as their active markers to ascertain their potential efficacy as Thai traditional medicine for wound healing.

## 2. Materials and Methods

### 2.1. Plant Materials

The selected medicinal plants: licorice (*G*. *glabra* L.), bullet wood (*M*. *elengi* L.), black cumin (*N*. *sativa* L.), Indian gooseberry (*P*. *emblica* L.), Indian nightshade (*S*. *indicum* L.), Thai nightshade (*S*. *trilobatum* L.), clove bud (*S*. *aromaticum* L.), and mangosteen (*G*. *mangostana* L.) were purchased from herbal shops in Thailand. The voucher specimens were deposited at the Herbarium of the Southern Center of Thai medicinal plants at the Faculty of Pharmaceutical Science, Prince of Songkla University, Songkhla, Thailand. The identities of the herbal materials were done by comparison with the authentic herbarium specimen (Table 1).

### 2.2. Chemicals and Reagents

Nitro blue tetrazolium chloride (NBT) and phorbol 12-myristate 13-acetate (PMA) were purchased from Sigma, Germany. Hanks' balanced salt solution (HBSS) was purchased from Gibco, USA. Lipopolysaccharide (LPS, from *Escherichia coli*) and 3-(4,5-dimethyl-2-thiazolyl)-2,5-diphenyl-2H-tetrazolium bromide (MTT) were from Sigma, USA. RPMI medium powder 1640 (RPMI 1640), DMEM medium powder, fetal bovine serum (FBS), penicillin-streptomycin (P/S), trypsin-EDTA, and trypan blue were purchased from Gibco, USA. Phosphate buffer saline (PBS) was from Amresco (USA) and other chemicals were from Sigma and Merck. *α*-Mangostin, asiaticoside and thymoquinone were purchased from Sigma, USA. Glycyrrhizin was purchased from Tokyo chemical industry (TCI), Singapore.

### 2.3. Preparation of Plant Materials

The plant materials were washed and sliced into small pieces then dried in a hot air oven at 50°C for 24 hrs.

### 2.4. Preparation of Plant Extracts

Each dried plant material was powdered and macerated at room temperature with 95%EtOH (300 g/500 ml) for 3 days and then filtered through Whatman No. 1 filter paper. The marc was remacerated twice. The filtrates were combined and evaporated to dryness. The ethanolic crude extracts from 8 plants were stored at −20°C until bioactivity testing. Aqueous extracts from 8 plant materials were obtained by decoction with distilled water for 15 min and then filtered through Whatman No. 1 filter paper. The decoction process was repeated twice with the marc. Filtrates were combined, lyophilized, and kept at −20°C until use.

### 2.5. Preparation of Extract for Biological Activity Testing

Ethanolic extracts were dissolved in sterile DMSO while aqueous extracts were dissolved in sterile distilled water and filtered through 0.22 *μ*m filter paper. All 16 extracts were stored at −20°C until use.

### 2.6. Measurement of Nitric Oxide Production Inhibition Assay from RAW 264.7 Cell Line

Murine macrophage leukemia cell line (RAW 264.7), purchased from American Type Culture Collection (ATCC, USA), was cultured in RPMI 1640 with 10% fetal bovine serum and 1% penicillin-streptomycin at 37°C in humidified atmosphere containing 5% CO_2_. The cells were harvested with 0.25% trypsin-EDTA by centrifugation at 1,500 rpm for 5 min and then diluted with fresh medium into a suspension at the concentration of 1 × 10^6^ cells/ml. Subsequently, 100 *μ*l of cell suspension was seeded into each of 96-well plates and incubated for 24 hrs to form a monolayer. After incubation, the supernatant was replaced with a fresh complete medium containing 10 ng/ml of LPS together with various concentrations of plant extracts and incubated for 24 hrs subsequently, a 100 *μ*l of supernatant liquid was transferred from the original plate (plate 1) to another plate (plate 2). The nitric oxide reduction from RAW 264.7 cell line was determined by measuring the accumulation of nitrite in the supernatant after the addition of 100 *μ*l of Griess reagent (0.1% naphthyl ethylenediamine dihydrochloride, 1% sulfanilamide, and 5% phosphoric acid in distilled water) and the content of developed azo dye was determined at 570 nm. Prednisolone was used as a positive control.

The viability of cells was determined by MTT assay after incubation with LPS and medicinal plant extracts for 24 hrs. MTT solution (5 mg/ml in PBS) was added to 96-well plates (plate 1) and incubated for 2 hrs, and then the supernatant was discarded. A solution of 0.04 M HCl in isopropanol was added to dissolve formazans formed in the cells and the absorbance measured at 570 nm [19]. The plant extract that showed viability of cell less than 70% was further diluted until 70% or higher viability was obtained, and its IC_50_ value was calculated.

### 2.7. Measurement of Superoxide Anion Radical Reduction Assay from HL-60 Cell Line

Human promyelocytic leukemia cell line (HL-60), purchased from the American Type Culture Collection (ATCC, USA), was cultured in RPMI 1640 with 10% fetal bovine serum and 1% penicillin-streptomycin at 37°C in humidified atmosphere containing 5% CO_2_. HL-60 cells were adjusted at the concentration of 5 × 10^5^ cells/ml and induced by 1.3% DMSO in RPMI 1640 for 6 days. In this way, HL-60 cells, differentiated into neutrophils, had smaller cell size with increased expression of the NADPH oxidase on the plasma membrane [20]. After differentiation, HL-60 cells were harvested by centrifugation at 1,500 rpm for 5 min, and then the supernatant was replaced with Hanks' balanced salt solution (HBSS) to form a suspension at the concentration of 5 × 10^6^ cells/ml. Subsequently, 200 *μ*l of cell suspension was added to the tube with 500 *μ*l of medicinal plant extract in Hanks' balanced salt solution (HBSS) and incubated at 37°C for 15 min. Then 50 *μ*l of phorbol 12-myristate 13-acetate (PMA) solution (2 mg/ml in DMSO) and 250 *μ*l of nitro blue tetrazolium (NBT) solution (1.25 mg/ml in HBSS) were added and incubated for 1 hr. The produced superoxide anion (O_2_^−^) was reduced with NBT solution giving the blue formazans, and then 2 ml of 1 M HCl at 4°C was added to the tube and centrifuged at 4,000 rpm for 10 min. The supernatant was discarded and the formazans were dissolved with DMSO and the absorbance was measured at 572 nm. Propyl gallate was used as a positive control.

The viability of the cells was determined by the MTT assay in parallel with the above NBT assay. The same procedure was executed without the addition of NBT; after 1 hr incubation, it was centrifuged at 1,500 rpm for 5 min and replaced the supernatant with HBSS containing MTT solution (5 mg/ml in PBS) and was further incubated for 2 hrs. The tubes were subjected to centrifugation at 4,000 rpm for 10 min; the supernatant was discarded before dissolving formazans with DMSO and the absorbance was determined at 570 nm [21]. The plant extract that showed viability of cell less than 70% of control was further diluted until 70% or higher viability was obtained, and its IC_50_ was calculated.

### 2.8. Determination of Proliferation with Fibroblast Cell Line

Murine embryonic fibroblast cell line (3T3-CCL92), purchased from the American Type Culture Collection (ATCC, USA), was cultured in DMEM with 10% fetal bovine serum and 1% penicillin-streptomycin at 37°C in humidified atmosphere containing 5% CO_2_. Cell proliferation assay, modified from that of Mosmann [22], was used in which the cells were harvested with 0.25% trypsin-EDTA and centrifugation at 1,500 rpm for 5 min. The supernatant was removed and the residue diluted with a fresh medium into a suspension at the concentration of 1 × 10^5^ cells/ml. Then 100 *μ*l of the cell suspension was seeded into each of the 96-well plates and incubated for 24 hrs. After incubation, the supernatant was replaced with medicinal plant extracts in basic growth media (DMEM with 0.5% fetal bovine serum and 1% penicillin-streptomycin). Asiaticoside was used as a positive control. After 24 hrs incubation, MTT solution (5 mg/ml in PBS) was added and further incubated for 2 hrs. The formazan was dissolved in DMSO and determined at 570 nm. The viability was calculated as percentage of proliferation [23] and plant extracts were selected for further study when their proliferation percentage was significantly more or equal to that of the control.

### 2.9. Determination of Wound Closure with Fibroblast Cell Line

After the viability assay results were evaluated, the medicinal plant extracts that exhibited equal or higher viability than the control were selected for the study of wound closure and spreading ability of murine embryonic fibroblast cells (3T3-CCL92). The scratch wound closure assay was done to investigate the wound contraction phase. The reduction in wound area was measured by ImageJ software and the percentage of recovery area and the acceleration rate relative to the control, for each dose and time, were calculated. The cells were harvested with 0.25% trypsin-EDTA and centrifugation at 1,500 rpm for 5 min after which the supernatant was replaced with a fresh medium to form a suspension at the concentration of 2 × 10^5^ cells/ml. Subsequently, 500 *μ*l of cell suspension was seeded into each of the 24-well plates and incubated for 24 hrs. to allow the cells to adhere into a monolayer. A linear wound was generated on the monolayer with a sterile 200 *μ*l plastic pipette tip with the wound width controlled at 550 ± 50 *μ*m. The cellular debris was removed by washing with phosphate buffer saline (PBS). The medicinal plant extract in basic media (DMEM with 0.5% fetal bovine serum and 1% penicillin-streptomycin) was added, and then the photographs of wound areas were taken at 0 and 24 hours [24]. DMEM medium with 0.2% DMSO was used as a control group with asiaticoside as a positive control. An extract concentration that showed maximum wound closure was chosen for the measurement of the acceleration rate by photographing every 3 hrs up to 24 hrs. The percentage of recovery area was calculated from each photograph and compared with the control group to elucidate wound closure ability of the plant extracts.

### 2.10. Selection Criteria of Active Markers

This study chose 4 bioactivities that would represent the wound healing potential of 16 plant extracts. These were production inhibition of nitric oxide, superoxide anion radical reduction, cell proliferation, and wound closure assays. The plant extracts that gave positive results with cell proliferation and wound closure assays were reviewed for their major active markers. These compounds were also tested for the above activities.

### 2.11. High-Performance Liquid Chromatography (HPLC) System

The HPLC (UV 1200 series, Agilent Technologies) equipped with an UV-detector was set at 253 nm. The chromatographic separation was performed at room temperature on an XDB-C18 analytical column (250 mm × 4.6 mm, USA) injection volume 10 *μ*l at low rate 1.0 ml/min. The mobile phase consisted of 0.1% v/v phosphoric acid (solvent A) and acetonitrile (solvent B). Total running time was 60 min and the gradient program was as follows: 5% B for 0–5 min, 5% B to 50% B for 35 min, 50% B to 95% B for 15 min, and 95% B to 5% B for 5 min. The compounds were quantified using Agilent lab software.

### 2.12. Statistical Analysis

All results are presented as mean ± SEM. Statistical analyses were performed with the standard program using one-way ANOVA with Bonferroni's multiple comparisons test at *p*=0.05, while the acceleration rate of recovery was determined by using linear regression to compare slopes of recovery rate at *p*=0.05.

## 3. Results

### 3.1. Yield of Herbal Extract by Different Extraction Methods

**Figure 1:**
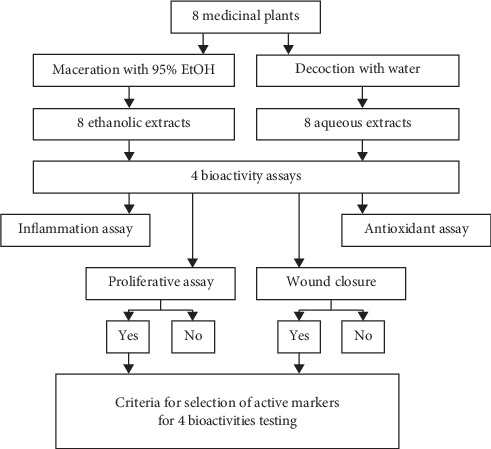
Flow chart of study.

The selected medicinal plants were extracted with two methods, maceration and decoction, producing 8 ethanolic and 8 aqueous extracts as shown in Table 1 and Figure 1. Among the ethanolic extracts obtained, *G*. *mangostana*, *N*. *sativa*, *S*. *aromaticum*, and *P*. *emblica* yielded greater than 10%, that is, 23.81, 22.07, 18.79, and 13.11%, respectively. *S*. *trilobatum*, *S*. *indicum*, *G*. *glabra*, and *M*. *elengi* produced medium-to-low yield, that is, 7.67, 6.77, 6.08, and 1.23%, respectively. With decoction, those giving higher yields were *P*. *emblica*, *S*. *trilobatum*, *G*. *glabra*, *N*. *sativa*, *S*. *aromaticum*, *S*. *indicum*, and *G*. *mangostana* with 22.11, 19.64, 15.33, 13.31, 13.15, 12.98, and 12.73%, respectively. *M*. *elengi* gave the lowest yield of 0.62%.

### 3.2. Production Inhibition of Nitric Oxide

 Anti-inflammatory activity was investigated through the production inhibition of NO in RAW264.7 murine macrophage cell line, using prednisolone as reference. Among 16 extracts tested, the ethanolic extract of *G*. *mangostana* exhibited the highest anti-inflammatory activity with the IC_50_ value of 23.97 ± 0.91 *μ*g/ml. The second and third active extracts were the ethanolic extracts of *G*. *glabra* and *M*. *elengi* with IC_50_ values of 46.35 ± 0.43 and 78.48 ± 4.46 *μ*g/ml, respectively. Other ethanolic extracts and all of the aqueous extracts did not show activity (IC_50_ values more than 100 *μ*g/ml). Later, thymoquinone and *α*-mangostin, the major compound of each plant which passed the criteria of selection (Figure 1), showed high activity against nitric oxide at the IC_50_ values of 1.55 ± 0.14 and 15.15 ± 0.14 *μ*g/ml, respectively, while glycyrrhizin had no activity (IC_50_ values more than 100 *μ*g/ml) (Table 2).

### 3.3. Reduction of Superoxide Anion

The results of antioxidant activity using HL-60 cell line are shown in Table 2. The ethanolic extract of *P*. *emblica*, the aqueous extract of *S*. *aromaticum*, and ethanolic extract of *G*. *mangostana* showed the highest activity against superoxide anion with IC_50_ values of 11.30 ± 0.66, 12.03 ± 0.79, and 13.97 ± 0.38 *μ*g/ml, respectively, which were not significantly different from propyl gallate (IC_50_ value of 6.48 ± 1.06 *μ*g/ml). Aqueous extracts of *P*. *emblica* and *G*. *mangostana* and ethanolic extracts of *S*. *indicum* and *G*. *glabra* exhibited high antioxidant activity with IC_50_ values of 14.59 ± 1.15, 16.89 ± 1.26, 26.83 ± 1.24, and 28.62 ± 1.91 *μ*g/ml, respectively. The extracts showing low activity were ethanolic extracts of *M*. *elengi* and *N*. *sativa* with IC_50_ value of 71.30 ± 3.29 and 71.54 ± 3.22 *μ*g/ml, respectively, while other extracts showed no antioxidant activity (IC_50_ value more than 100 *μ*g/ml). The major compounds, *α*-mangostin and thymoquinone, which passed the criteria of selection (Figure 1) showed high activity against superoxide anion with the IC_50_ values of 2.65 ± 0.52 and 9.56 ± 1.11 *μ*g/ml, respectively, while glycyrrhizin displayed moderate activity against superoxide anion with the IC_50_ values of 40.85 ± 2.30 *μ*g/ml.

### 3.4. Increasing Fibroblast Proliferation

The selection of the plant extracts for the proliferation of 3T3-CCL92 murine embryonic fibroblast cell line was based on the results of viability assay (Table 3). The ethanolic extracts of *G*. *mangostana*, *G*. *glabra*, *α*-mangostin, glycyrrhizin, and thymoquinone were toxic with IC_50_ values of 44.63 ± 0.30, 52.58 ± 1.45, 5.31 ± 0.02, 42.71 ± 0.59, and 7.29 ± 0.11 *μ*g/ml, respectively. Other ethanolic and aqueous extracts were not toxic. Appropriate concentrations of the ethanolic extracts, aqueous extracts, and pure compounds were used accordingly. The ethanolic extracts of *G*. *mangostana*, *G*. *glabra*, and *N*. *sativa* could increase the proliferation to the maximum of 52.68 ± 1.99, 52.13 ± 3.95, and 15.47 ± 0.87%, respectively. These were significantly different from that of negative control. Interestingly, the result of *N*. *sativa* extract was comparable to that of asiaticoside, the positive control, with the maximum proliferation value of 13.71 ± 0.51%. Among the pure compounds tested, *α*-mangostin gave maximal proliferation of 26.72 ± 2.21%, while glycyrrhizin and thymoquinone did not promote cell proliferation (Table 3). It should be noted that the ethanolic extracts of *G*. *mangostana* and *G*. *glabra* and *α*-mangostin promoted significantly greater cell proliferation than the positive control. The negative results were not presented (Table 3).

### 3.5. Increasing Wound Closure of Fibroblast

**Figure 2:**
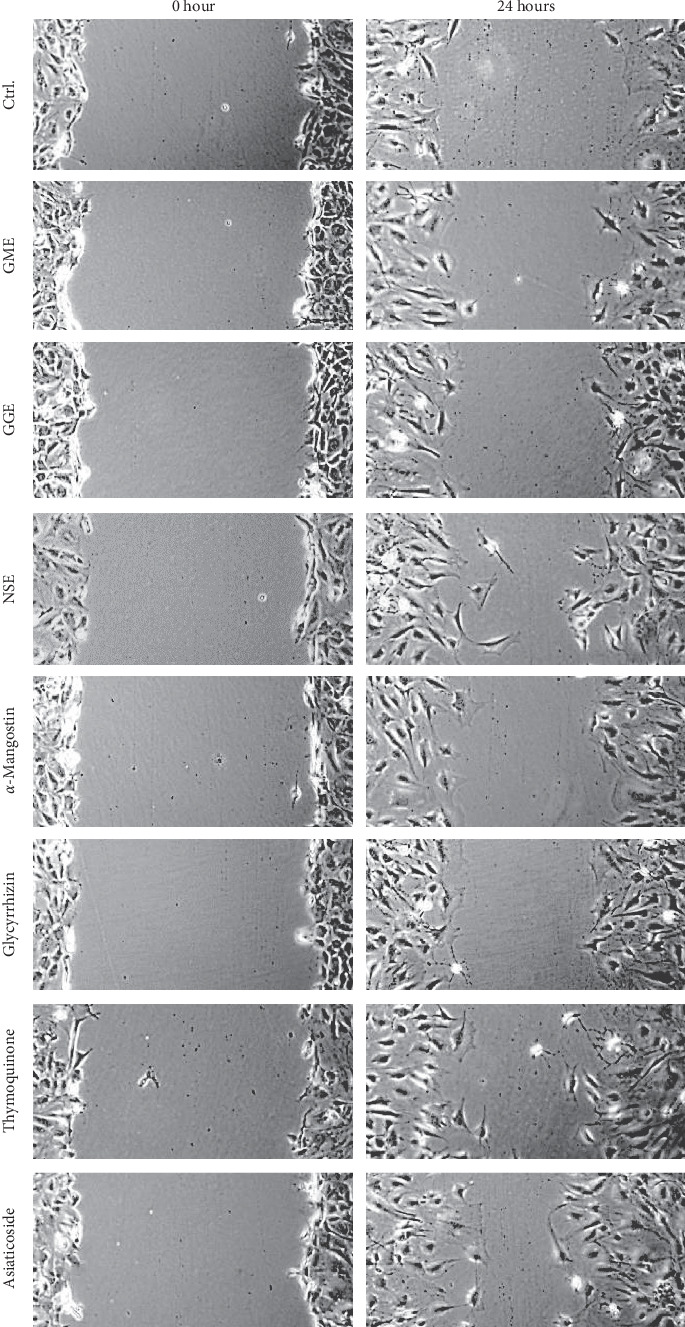
Recovery area of 3T3-CCL92 cell line at 0 and 24 hours with ethanolic extracts of *Garcinia mangostana* (GME), *Glycyrrhiza glabra* (GGE), *Nigella sativa* (NSE), and their major compounds, *α*-mangostin, glycyrrhizin, and thymoquinone, at the concentrations of 2.5, 10, 100, 1, 10, and 0.1 *μ*g/ml, respectively. 0.2% DMSO in basic media and asiaticoside at the concentration of 100 *μ*g/ml were used as negative control (Ctrl.) and positive control, respectively.

The wound closure of 3T3-CCL92 cell line can promote wound healing process, the results showed that three extracts could increase the recovery rate (Table 3 and Figure 2). The ethanolic extracts of *G*. *mangostana*, *G*. *glabra*, and *N*. *sativa* displayed maximum cell recovery area with the values of 48.42 ± 1.26, 50.72 ± 0.78, and 60.60 ± 2.31% at the acceleration rates of 2.02 ± 0.03, 2.12 ± 0.03, and 2.65 ± 0.05% per hour, respectively. Their major compounds, namely, *α*-mangostin, glycyrrhizin, and thymoquinone, showed the promotion of the recovery area with the values of 51.63 ± 1.02, 51.87 ± 1.28, and 57.88 ± 0.34% at the acceleration rates of 2.18 ± 0.03, 2.18 ± 0.02, and 2.35 ± 0.05% per hour, respectively.

In this regard, the results obtained from these three plant extracts and their major markers were significantly different from the negative control. Thymoquinone promoted the acceleration as much as asiaticoside, the positive control, while the ethanolic extract of *N*. *sativa* promoted significantly greater acceleration of wound recovery than the positive control with the value of 58.80 ± 1.11% and the acceleration rate of 2.42 ± 0.03% of recovery area per hour. However, all aqueous extracts and other plant extracts did not promote wound closure activity. The negative results were not presented (Table 3).

### 3.6. High-Performance Liquid Chromatography for Selected Markers

**Figure 3:**
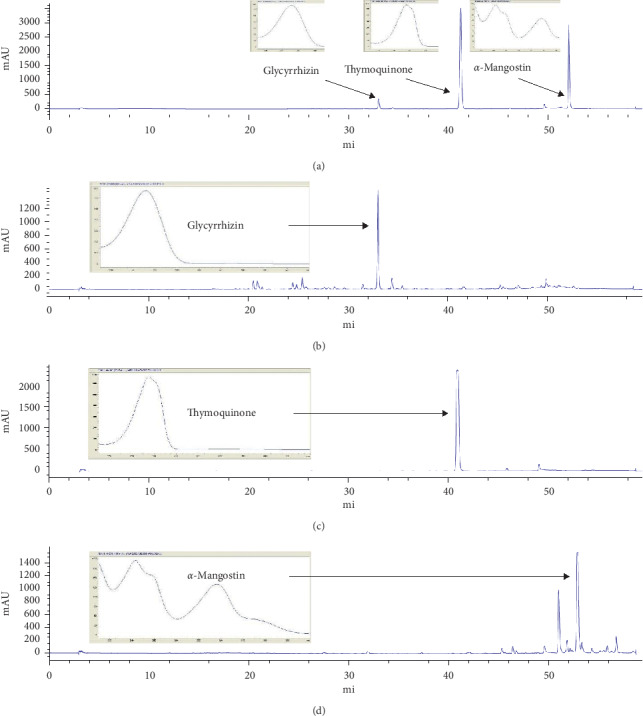
HPLC chromatograms of 3 markers mixture (a), glycyrrhizin in *G*. *glabra* (b), thymoquinone in *N*. *sativa* (c), and *α*-mangostin in *G*. *mangostana* (d) with UV spectra.

HPLC with gradient elution was used to detect the active marker of each selected medicinal plant, the HPLC chromatogram is shown in Figure 3. Glycyrrhizin, thymoquinone, and *α*-mangostin appeared at 33.02, 40.92, and 52.88 min, respectively. The identity of each peak was confirmed by its UV spectrum which was superimposable with that of standard.

## 4. Discussion

Eight Thai medicinal plants were selected from Mukkharoka scripture, the National List of Essential Medicines of Thailand, and traditional usages. These were *Nigella sativa* seed, *Glycyrrhiza glabra* root, *Phyllanthus emblica* fruits, *Solanum indicum* fruits and *Solanum trilobatum* fruits, *Garcinia mangostana* pericarp, *Mimusops elengi* bark, and clove bud (*Syzygium aromaticum*). Four *in vitro* studies were chosen to demonstrate whether their ethanolic and aqueous extracts could influence wound healing. At the first stage of wound healing, inflammation occurs; this involves RNS and ROS from our immune cells. These free radicals are essential for ridding of bacteria but it could also cause damage to cells surrounding the wound. In this regard, the tests for anti-inflammation and antioxidant were used. At the second stage of wound healing fibroblast cell proliferation commences and the wound reduces its size. To demonstrate these activities of the 16 plant extracts, fibroblast cell proliferation and scratch wound closure assays were chosen.

The results showed that ethanolic extracts of *G*. *mangostana* and *G*. *glabra* could reduce superoxide anion, inhibit nitric oxide production, promote fibroblast proliferation, and accelerate wound closure. These two extracts would be useful for the first and second stages of wound healing as described above. Although the ethanolic extract of *N*. *saliva* showed the lowest activity against superoxide anion and no activity against nitric oxide, it showed the highest wound closure activity among all extracts and asiaticoside, the reference compound. *N*. *sativa* extract would be beneficial at the second stage of wound healing. For this reason, these three ethanolic plant extracts had passed the selection criteria in testing their markers for these activities. These markers were *α*-mangostin for *G*. *mangostana*, glycyrrhizin for *G*. *glabra*, and thymoquinone for *N*. *sativa*.

Concerning *G*. *mangostana* ethanolic extract, the results showed that IC_50_ values of antioxidant and anti-inflammation activities were 13.97 ± 0.38 and 23.97 ± 0.91 *μ*g/ml, respectively. Its marker, *α*-mangostin, also showed stronger activities with the IC_50_ values of 2.65 ± 0.52 and 15.15 ± 0.14 *μ*g/ml, respectively. Both extract and marker could promote cell proliferation to the maximal values of 52.68 ± 1.99 and 26.72 ± 2.21%, respectively. The acceleration of wound closure was also increased at the rates of 2.02 ± 0.03 and 2.18 ± 0.03% per hour, respectively. Our results were in accordance with previous studies which showed that the ethanolic extract of *G*. *mangostana* and its major compound, *α*-mangostin, exerted strong anti-inflammatory activity through the production inhibition of nitric oxide and PGE_2_ [25] and also showed free radicals (superoxide anion and DPPH) inhibitions [26]. Another *in vivo* study also showed wound healing activity of *G*. *mangostana* extract [27].

The ethanolic extract of *G*. *glabra* inhibited superoxide anion and nitric oxide production with the IC_50_ values of 28.62 ± 1.91 and 46.35 ± 0.43 *μ*g/ml, respectively. Its marker, glycyrrhizin, exhibited antioxidant activities with the IC_50_ value of 40.85 ± 2.30 *μ*g/ml but has no activity against nitric oxide production inhibition. *G*. *glabra* extract also promoted proliferation to the maximal values of 52.13 ± 3.95 and accelerated wound closure at the rate of 2.12 ± 0.03% per hour. Glycyrrhizin, on the contrary, had no activity on promoting fibroblast proliferation but could increase the acceleration of wound closure at the rate of 2.18 ± 0.02% recovery area per hour. These results indicated that the ethanolic extract of *G*. *glabra* was active for all activities but not entirely due to glycyrrhizin. Our results were in agreement with a recent study on *G*. *glabra* extract against superoxide anion and nitric oxide at IC_50_ values of 38.4 and 62.1 *μ*g/ml, respectively [28]. Another previous study on the treatment of open wounds in rabbits using creams containing *G*. *glabra* extract also showed better healing than dexpanthenol cream [29].

The ethanolic extract of *N*. *sativa* exhibited no activity against production inhibition of nitric oxide (IC_50_ value > 100 *μ*g/ml) and low activity against superoxide anion with the IC_50_ value of 71.54 ± 3.22 *μ*g/ml. However, it was able to increase proliferation to the maximum of 15.47 ± 0.87% and gave the highest wound closure activity with the acceleration rates of 2.65 ± 0.05% of recovery area per hour. Its marker, thymoquinone, showed high activities against nitric oxide and superoxide anion with the IC_50_ value of 1.55 ± 0.14 and 9.56 ± 1.11 *μ*g/ml which were not significantly difference from reference compounds (prednisolone and propyl gallate). It also promoted wound closure activity with the acceleration rates of 2.35 ± 0.05% of recovery area per hour without any proliferative promoting activity. The results showed that the ethanolic extract of *N*. *sativa* gave good results with proliferation and wound closure activities while thymoquinone exhibited high activities as antioxidant and anti-inflammation through production inhibition of superoxide anion and nitric oxide activities. Therefore, the ethanolic extract of *N*. *sativa* could be good choice for the wound at the second phase. Our result was in accordance with a previous study that reported nitric oxide inhibition by *N*. *sativa* extract at the concentration higher than 100 *μ*g/ml [7, 30].

Our results suggested that ethanolic extracts of *G*. *mangostana*, *G*. *glabra*, and *N*. *sativa* may be applied to treat the wound in various phases. They can be divided into two groups. The first group consists of the ethanolic extracts of *G*. *mangostana* and *G*. *glabra* which can be applied together with the saline washing solution to remove foreign bodies and bacteria. This is due to the fact that the ethanolic extracts of *G*. *mangostana* and *G*. *glabra* can reduce inflammation and protect the wound surrounding cells. They have been shown to effectively inhibit *Staphylococcus aureus* and *Staphylococcus aureus* MRSA with the Minimal Inhibitory Concentration (MIC) values of 9.8 and 4.9 *μ*g/ml, respectively [31]. The second group consists of the ethanolic extract of *N*. *sativa* which can be applied on open wounds or wounds that are not completely close, to accelerate wound recovery. Glycyrrhizin and thymoquinone enhanced wound closure without any cell proliferation activities and may be beneficial in the second phase of wound treatment as well as *α*-mangostin. Interestingly, *G*. *mangostana* and *N*. *sativa* yielded high percentage of their ethanolic extracts which made them more attractive for future utilization.

## 5. Conclusions

Our findings demonstrated that 3 commonly used Thai medicinal plants are effective for wound treatment and supported their uses in Thai traditional medicine. Their effectiveness can be scientifically explained in terms of the activities of these plant extracts and their active markers that promote wound healing through the inhibition of nitric oxide and superoxide anions and through increasing fibroblast cell proliferation and accelerating wound closure. Further studies are necessary to explain the wound healing property of these herbs and to develop them into a potential wound healing agent.

## Figures and Tables

**Table 1:** Ethnobotanical data, specimen voucher number, and percentage of yield by maceration and decoction methods.

No.	Scientific name	Common name	Family name	Part used	Specimen voucher number	% yield	
						Maceration	Decoction
1	*Garcinia mangostana* L.	Mangosteen	Clusiaceae	Pericarp	SKP 214 09 13 01	23.81	12.73
2	*Glycyrrhiza glabra* L.	Licorice	Fabaceae	Root	SKP 072 07 07 01	6.08	15.33
3	*Mimusops elengi* L.	Bullet wood	Sapotaceae	Bark	SKP 171 13 05 01	1.23	0.62
4	*Nigella sativa* L.	Black cumin	Ranunculaceae	Seed	SKP 160 14 19 01	22.07	13.31
5	*Phyllanthus emblica* L.	Indian gooseberry	Euphorbiaceae	Dry fruit	SKP 071 16 05 01	13.11	22.11
6	*Solanum indicum* L.	Indian nightshade	Solanaceae	Dry fruit	SKP 180 19 09 01	6.77	12.98
7	*Solanum trilobatum* L.	Thai nightshade	Solanaceae	Dry fruit	SKP 180 19 20 01	7.67	19.64
8	*Syzygium aromaticum* L.	Clove	Myrtaceae	Bud	SKP 123 19 01 01	18.79	13.15

**Table 2:** Effectiveness of medicinal plant extracts on HL-60 and RAW 264.7 cell lines in inhibiting superoxide anions and nitric oxide.

No.	Type of extract	Medicinal plant extracts and chemical compounds	NBT		NO	
			IC_50_ ± SEM (*μ*g/ml)	*p* value	IC_50_ ± SEM (*μ*g/ml)	*p* value
1	—	Prednisolone	—	—	1.30 ± 0.05	(Ref)
2	—	Propyl gallate	6.48 ± 1.06	(Ref)	—	—
3	Ethanolic	*Phyllanthus emblica* L.	11.30 ± 0.66	0.630	>100	—
4	Aqueous	*Syzygium aromaticum* L.	12.03 ± 0.79	0.355	>100	—
5	Ethanolic	*Garcinia mangostana* L.	13.97 ± 0.38	0.062	23.97 ± 0.91	<0.001
6	Aqueous	*Phyllanthus emblica* L.	14.59 ± 1.15	0.039	>100	—
7	Aqueous	*Garcinia mangostana* L.	16.89 ± 1.26	0.005	>100	—
8	Ethanolic	*Solanum indicum* L.	26.83 ± 1.24	<0.001	>100	—
9	Ethanolic	*Glycyrrhiza glabra* L.	28.62 ± 1.91	<0.001	46.35 ± 0.43	<0.001
10	Ethanolic	*Mimusops elengi* L.	71.30 ± 3.29	<0.001	78.48 ± 4.46	<0.001
11	Ethanolic	*Nigella sativa* L.	71.54 ± 3.22	<0.001	>100	—
12	—	*α*-Mangostin	2.65 ± 0.52	0.203	15.15 ± 0.14	<0.001
13	—	Thymoquinone	9.56 ± 1.11	0.341	1.55 ± 0.14	0.796
14	—	Glycyrrhizin	40.85 ± 2.30	<0.001	>100	<0.001

Note: plant extracts with negative results were not shown.

**Table 3:** Effectiveness of medicinal plant extracts on cell viability, proliferation, and wound closure activities on 3T3 CCL-92 cells.

No.	Type of extract	Medicinal plant extracts and chemical compounds	Toxicity IC_50_ ± SEM (*μ*g/ml)	Proliferation			Wound closure		
				Conc. (*μ*g/ml)	% ± SEM	*p* value	% ± SEM	Acceleration (% recovery/hour)	*p* value
1	—	0.2% DMSO in DMEM	>100	—	0 ± 0	<Ref.>	39.67 ± 1.15	1.57 ± 0.03	<Ref.>

2	Ethanolic	*Garcinia mangostana* L.	44.63 ± 0.30	1	2.01 ± 0.40	0.953	40.21 ± 0.76	—	—
				2.5	23.38 ± 2.42	<0.001	48.42 ± 1.26	2.02 ± 0.03	<0.001
				5	52.68 ± 1.99	<0.001	42.94 ± 1.10	—	—
				10	33.99 ± 5.08	<0.001	32.75 ± 2.51	—	—

3	Ethanolic	*Glycyrrhiza glabra* L.	52.58 ± 1.45	1	4.51 ± 1.58	0.453	43.79 ± 1.36	—	—
				5	12.08 ± 1.35	0.011	47.73 ± 0.61	—	—
				10	22.38 ± 2.23	<0.001	50.72 ± 0.78	2.12 ± 0.03	<0.001
				25	52.13 ± 3.95	<0.001	35.54 ± 1.94	—	—

4	Ethanolic	*Nigella sativa* L.	>100	1	2.80 ± 0.77	0.058	42.36 ± 0.49	—	—
				10	5.27 ± 0.56	0.01	41.44 ± 0.41	—	—
				50	12.45 ± 0.91	<0.001	51.67 ± 1.33	—	—
				100	15.47 ± 0.87	<0.001	60.60 ± 2.31	2.65 ± 0.05	<0.001

5	—	*α*-Mangostin	5.31 ± 0.02	0.01	0.96 ± 0.27	0.948	39.73 ± 1.92	—	—
				0.1	1.30 ± .76	0.870	42.70 ± 1.44	—	—
				1	5.05 ± 1.48	0.052	51.63 ± 1.02	2.18 ± 0.03	<0.001
				2.5	26.72 ± 2.21	<0.001	36.76 ± 2.52	—	—

6	—	Glycyrrhizin	42.71 ± 0.59	0.1	0.59 ± 0.96	0.962	40.79 ± 0.66	—	—
				1	−0.26 ± 0.93	0.998	41.57 ± 2.73	—	—
				5	−0.04 ± 0.66	0.999	49.99 ± 0.65	—	—
				10	−1.47 ± 1.14	0.567	51.87 ± 1.28	2.18 ± 0.02	<0.001

7	—	Thymoquinone	7.29 ± 0.11	0.01	−0.63 ± 1.12	0.994	45.68 ± 0.55	—	—
				0.1	−0.11 ± 1.50	0.999	57.88 ± 0.34	2.35 ± 0.05	<0.001
				1	−0.74 ± 0.76	0.989	50.10 ± 0.72	—	—
				2.5	−2.33 ± 1.67	0.655	38.81 ± 0.71	—	—

8	—	Asiaticoside	>100	1	1.75 ± 1.31	0.614	43.55 ± 1.70	—	—
				10	3.35 ± 1.39	0.145	42.91 ± 0.90	—	—
				50	8.24 ± 1.32	0.001	54.85 ± 1.19	—	—
				100	13.71 ± 0.51	<0.001	58.80 ± 1.11	2.42 ± 0.03	<0.001

## Data Availability

The data used to support the findings of this study are included within the article.
